# Probabilistic human health risk assessment of PM_2.5_ exposure in communities affected by local sources and gold mine tailings

**DOI:** 10.3389/fpubh.2025.1515009

**Published:** 2025-07-28

**Authors:** Nomsa Duduzile Lina Thabethe, Tafadzwa Makonese, Daniel Masekameni, Derk Brouwer

**Affiliations:** ^1^Department of Environmental Sciences, University of South Africa, Florida, South Africa; ^2^Health Sciences Faculty, School of Public Health, University of the Witwatersrand, Johannesburg, South Africa; ^3^Department of Development Studies, University of South Africa, Pretoria, South Africa

**Keywords:** fine particulate matter, exposure assessment, Monte Carlo simulations, sensitivity analysis, mining activities

## Abstract

Epidemiological studies have found that exposure to fine particulate matter (PM_2.5_) poses potential human health risks, including respiratory, cardiovascular and cerebrovascular diseases. This study aimed to assess the potential human health risks associated with exposure to PM_2.5_ in the eMbalenhle community which is near gold mine Tailings Storage Facilities (TSFs). Ambient PM_2.5_ concentrations were measured for 1 year (from February 2022 to February 2023) using the Clarity Node-S low-cost monitor (LCM). The United States Environmental Protection Agency (USEPA) equations were used to estimate the carcinogenic and non-carcinogenic health risks associated with exposure to PM_2.5_ in toddlers, children, adults and the older adult. Lastly, a probabilistic Human Health Risk Assessment (HHRA) model, which employs Monte Carlo simulations (MCS), was applied to assess the sensitivity and uncertainty risks. The annual PM_2.5_ Geometric Mean (GM) concentration were 17, with a Standard Deviation of (SD) of 10.4 and a Geometric Standard Deviation (GSD) of 1.69 μg/m^3^. This was below the South African annual National Ambient Air Quality Standards (NAAQS) of 20 μg/m^3^. However, this concentration exceeded the World Health Organization (WHO) guidelines and the USEPA annual limit values of 5 and 9 μg/m^3^, respectively. For the WHO guidelines, South African and USEPA NAAQS, the HQ was highest at the 95th percentile for all subgroups. For the South African NAAQS, the HQ was estimated to be 0.9 for all subgroups, indicating safe levels. When utilizing the USEPA NAAQS, a value of 2.5 was reported, while the WHO guidelines recorded the highest HQ of 3.5, indicating unsafe levels. This demonstrated that the SA NAAQS underestimated exposure to PM_2.5_ concentrations. Probabilistic HHRA assessed potential cancer risk (CR) due to continuous exposure to PM_2.5_ concentrations. For both male and female elders, the CR was approximately 1 in 10, meaning that about 100,000 out of 1,000,000 exposed elders were at an increased risk of developing cancer over their lifetime. The study recommends revising the current South African PM_2.5_ NAAQS to adopt more stringent measures and align them to international benchmarks to safeguard the public from adverse health effects due to PM_2.5_ exposure.

## 1 Introduction

Ambient air pollution is regarded as a significant threat to human health. According to the World Health Organization (1), the combined effects of ambient air pollution and household air pollution account for 6.7 million premature deaths globally. Fine particulate matter, with an aerodynamic diameter of 2.5 μm (PM_2.5_), has been associated with many adverse health outcomes (2–5) because it can penetrate deeper into the alveolar regions of the lungs (6, 7). Human health risks of exposure to PM_2.5_ extend beyond respiratory diseases to include cerebrovascular and cardiovascular diseases (8, 9), lung cancer, cardiopulmonary mortality, stroke, asthma, arrhythmia (10, 11). Exposure to PM_2.5_ affects individuals differently based on their health status, age (12), gender and duration of exposure (13).

Vulnerable population sub-groups are likely to develop diseases due to lifetime PM_2.5_ exposure to PM_2.5_ (14). These groups include pregnant women, infants and children. Children breathe faster than adults because smaller lungs require more frequent breaths to meet oxygen demands (15, 16). Exposure to high PM_2.5_ in these groups can affect how their lungs develop over time, increasing their chances of developing lung diseases (17). Exposure to PM_2.5_ during pregnancy can impact fetal development and may have long-term health consequences (18, 19). The physiological development, high Inhalation Rate (IR), Body Weight (BW), Exposure Duration (ED) and Exposure Frequency (EF) are some factors that make individuals vulnerable to the adverse health risks of PM_2.5_ exposure (20). Organs such as the lungs and heart undergo significant maturation over time (21). Exposure to PM_2.5_ during these stages of organ development can lead to long-term health risks (22). The older adult are more at risk of developing adverse health risks due to weakened respiratory and cardiovascular systems (23). Furthermore, pre-existing medical conditions, including asthma, heart disease and chronic obstructive pulmonary disease (24), increase their vulnerability to the harmful effects of pollution (25).

Fine particulate matter (PM_2.5_) is emitted from natural and anthropogenic sources (26). Natural sources of PM_2.5_ include forest fires and volcanic eruptions. Forest fires emit huge amounts of PM_2.5_ from the combustion of vegetation and biomass (27). Volcanic eruptions release ash and PM_2.5_ into the atmosphere. Anthropogenic sources of PM_2.5_ include industrial processes, domestic fuel burning, mining operations and agricultural activities. Burning of wood, coal and other solid fuels for residential heating and cooking also emits PM_2.5_ (28). Gold mine tailings storage facilities (TSFs) are also a potential source of PM_2.5_ emissions. The TSFs are designed to store waste generated during mineral extraction and processing. These tailings consist of crushed rocks, particles and residuals of chemicals left over from the gold extraction process (29).

PM_2.5_ comprises a complex mixture of biological and chemical components (30). The chemical and biological components of PM_2.5_ may originate from various sources (31). The biological components of PM_2.5_ include bacteria, fungi, viruses and pollen (32). The chemical components of PM_2.5_ include organic compounds (33), inorganic compounds (34) and trace elements (35). The physicochemical properties of fine particulate matter influence their toxicity and health impacts. Particles with larger surface areas and reactive chemical elements, can induce oxidative stress and inflammation, resulting in adverse respiratory and cardiovascular outcomes (36).

The assessment of exposure to PM_2.5_ is a complex process. It requires information about the sources, site selection and data quality assurance (37). PM_2.5_ concentrations vary spatially due to differences in emission sources, meteorological conditions, and topographical features. Furthermore, PM_2.5_ concentrations can fluctuate over time due to diurnal patterns and seasonal variations (38). Human health risks can be assessed by applying the HHRA model (39), which estimates the likelihood of adverse human health risks associated with PM_2.5_ exposure (40). Most studies have used deterministic HHRA models to assess potential human health risks (41–43). A deterministic HHRA model uses single-point estimates for input parameters to calculate specific risk values. One of the advantages of deterministic risk assessment is its simplicity. The disadvantage is its inability to account for the variability among individuals and the uncertainties in environmental data (44).

This study used a probabilistic HHRA model to estimate the potential human health risks of continuous exposure to PM_2.5_ concentrations. The probabilistic HHRA model was chosen because it uses statistical techniques to account for variability and uncertainty in risk estimates (45). Furthermore, the model employs Monte Carlo simulations to generate a distribution of risk estimates based on random sampling from the probability distributions of input parameters (46). The advantage of probabilistic HHRA provides a more complete picture of potential health risks by estimating the range and likelihood of different outcomes. The disadvantage is that it requires detailed data on the distribution of input parameters (47).

This study introduces a novel approach by applying a probabilistic Human Health Risk Assessment (HHRA) to evaluate the health risks associated with PM_2.5_ exposure in eMbalenhle, a community near gold mine tailings storage facilities (TSFs). While most existing studies rely on deterministic risk assessments that often fail to capture the inherent uncertainties and variability in PM_2.5_ exposure (39, 41, 43), this study addresses these limitations by adopting a probabilistic framework. Furthermore, the application of this method to communities affected by gold mine TSFs remains limited in the current literature. Importantly, this is the first study to assess human health risks in eMbalenhle using PM_2.5_ data collected from low-cost monitors (LCMs), offering a cost-effective and practical strategy for air quality and risk assessment in resource-constrained settings. The findings of this study will contribute to Sustainable Development Goal 3 (SDG 3). SDG 3 aims at ensuring good health and well-being for all. Monitoring the ambient PM_2.5_ contributes to achieving Target 3.9 of SDG 3, which addresses environmental pollution and its health consequences (48).

## 2 Methods and materials

### 2.1 Study design

The study followed a cross-sectional design following quantitative data collection and analysis methods.

### 2.2 Study area

eMbalenhle (−26°550613°S; 29.078937°E) is in the Govan Mbeki Municipality, Mpumalanga, South Africa. eMbalenhle has a population of 118,889 people with an annual growth of 2.5% (49). eMbalenhle is known for its gold and coal mining operations, which are potential sources of PM_2.5_. The area falls within the Highveld Priority Area (HPA). This area was declared an air pollution “hotspot” in terms of Section 18 (5) of the National Ambient Air Quality Act, Act 39 of 2004 (50). The town is close to industries, open-cast mines, and coal-fired power stations. As of the 16th of July 2024, the real-time Air Quality Index (AQI) displayed a PM_2.5_ concentration in eMbalenhle of 155 μg/m^3^, about 31 times above the WHO annual air quality Guideline value (51). This further indicated an unhealthy situation for vulnerable groups living in the area (52).

### 2.3 Data collection and analysis

The PM_2.5_ emissions were measured at the Sasol Recreation Centre (−26°550613°S; 29.078937°E) in eMbalenhle for 1 year (February 2022 to February 2023) using Clarity Node-S low-cost monitors (LCM) (53). These LCM measured PM_2.5_ concentrations every 15 min in micrograms per cubic meter (μg/m^3^). The ambient PM_2.5_ data monitored by the government ambient air monitoring station (also collocated at the Sasol Community Recreation Centre) for the same period was used as reference data. The reference data was used to compare and validate the data collected with the LCM. Both data sets (LCM and reference data) underwent quality control to ensure that the monitored and reference data were not erroneous (54). The meteorological data for the same period (February 2022 to February 2023) was obtained from the South African Air Quality Information System website (55). The average annual PM_2.5_ concentrations were calculated in a Microsoft Excel sheet and compared with the SA NAAQS, USEPA NAAQS and the WHO Guidelines. The PM_2.5_ annual Reference Concentrations (Rfc) values used in this study are given in Table 1.

**Table 1:** Local and international ambient PM_2.5_ annual reference concentration values.

Standard	Reference value	Description
	(μg/m^3^)	
NAAQS (SA)	20	National Standard
US EPA (USA)	9	National Standard
WHO	5	Guidelines

### 2.4 Probabilistic human health risk assessment

The population of eMbalenhle was divided into eight sub-groups because of the differences in exposure duration and development stages. The eight sub-groups included male and female toddlers (1–2 years) because their bodies are still developing and they have low IR; male and female children (6–11 years) because their bodies have developed and they have increased IR; male and female adults (21–60 years) because their bodies are fully developed and they have a much higher IR as compared to the toddlers and children; and male and female elders (61–70 years) because their health is weakening (56).

#### 2.4.1 Monte Carlo simulation

Monte Carlo Simulation (MCS) was employed to generate samples from probability distributions, using an Oracle Crystal Ball spreadsheet-based application for predictive modeling. parameters, including PM_2.5_ concentrations (C_air_), EF, Exposure Time (ET), ED, and Average Time (AT), were considered as variables in the model. The ET, EF, ED and AT parameters were allocated triangular, uniform and normal distributions. The C_air_ was allocated a lognormal distribution. The model used Geometric Mean (GM) and Geometric Standard Deviation (GSD) of the annual PM_2.5_ concentrations as C_air_.

Table 2 lists all the variables used in the probabilistic HHRA model. The annual GM and GSD PM_2.5_ concentrations used in the model were measured from February 2022 to February 2023. The ET was assumed to be 16 (minimum), 18 (mode), and 20 (maximum) hours for toddlers and children (16, 22, 24), and 20, 22, 24 for adults and elders. The EF was estimated based on the assumption that all population sub-groups leave the area for a maximum of 1 month, which translates to 335 days per year and a minimum of 2 weeks for vacation (i.e., the minimum EF is 350 days per year). The worst-case scenario assumed that all population subgroups are exposed 365 days per year. The AT for CR was based on chronic exposure during the lifetime. Lifetime values were obtained from the life-expectancy data according to the national census and population estimates (57). Population estimates for life expectancy from 2002 to 2022 were used to compute the life expectancy arithmetic mean and the standard deviation values used in our model.

**Table 2:** Variables used in the probabilistic human health risk assessment model.

Parameter	Unit	Distribution	Value	Source
C_air_	μg/m^3^	Lognormal	GM 17 (GSD 1.69)	PM_2.5_ Annual Average Concentrations (53)
ET (Toddler)	hours/day	Triangle	Min-mode-max 18–22–24	(Section 2.3.1)
ET (Child)	hours/day	Triangle	16–22–24	(Section 2.3.1)
ET (Adult)	hours/day	Triangle	20–22–24	(Section 2.3.1)
ET (Elder)	hours/day	Triangle	20–22–24	(Section 2.3.1)
AT (CR) – All Males	years	Normal	57.26 ± 4.71	(49)
AT (CR) – All Females	years	Normal	62.19 ± 5.53	(49)
ED (Toddler)	years	Uniform	Min-max 1–2	(Section 2.3.1)
ED (Child)	years	Uniform	6–11	(Section 2.3.1)
ED (Adult)	years	Uniform	21–60	(Section 2.3.1)
ED (older adult)	years	Uniform	61–70	(Section 2.3.1)
EF	days/year	Triangle	335–350–365	(Section 2.3.1)
Rfc (Annual PM_2.5_ -SA NAAQS)	μg/m^3^	Not Applicable	20	(50)
Rfc (Annual PM_2.5_ – WHO Guidelines)	μg/m^3^	Not Applicable	5	(51)
Rfc (Annual PM_2.5_ US EPA NAAQS)	μg/m^3^	Not Applicable	9	(53)
IUR	μg/m^3^	Not Applicable	0.008	(63)
C_air-adj (HQ)_	μg/m^3^	Not Applicable	Calculated	Output
C_air-adj (CR)_	μg/m^3^	Not Applicable	Calculated	Output
HQ (SA-NAAQS)	Unitless	Not Applicable	Calculated	Output
HQ (WHO Guidelines)	Unitless	Not Applicable	Calculated	Output
HQ (US EPA NAAQS)	Unitless	Not Applicable	Calculated	Output
Cancer Risk	Unitless	Not Applicable	Calculated	Output

Once the distributions of the variables were determined as normal, lognormal or triangle, the model randomly selected a value for each variable and calculated the risk. In MCS, each calculation is called an iteration, and a set of iterations is called a simulation (58). The probabilistic health risk distribution was obtained by running 10,000 iterations (59, 60).

The HQ was calculated using Equation 1 for each population sub-group to assess the non-carcinogenic health risks associated with exposure to PM_2.5_.

(1) $$\mathrm{HQ}=C_{\mathrm{air}-\mathrm{adj}}/\mathrm{Rfc}$$

Where:

HQ is unitless, representing the ratio of the concentration of PM_2.5_ in the air (C_air_) to the Reference Concentration (Rfc), (61). Since the Rfc assumes continuous exposure 24 h/7 days a week and 52 weeks/year, the measured concentration of PM_2.5_ must be adjusted to the actual duration of the exposure. The adjusted PM_2.5_ concentrations (C_air-adj_) were estimated using Equation 2 derived from the USEPA (61).

(2) $$C_{\mathrm{air}-\mathrm{adj}}=C_{\mathrm{air}}\times (\mathrm{ET}\times 1/24\;\text{hours})\times (\mathrm{EF}\times 1/365)\times \mathrm{ED}/\mathrm{AT}$$

Where:

- C_air_ = Concentration of contaminant in air (μg/m^3^)
- ET = Exposure time (hours/day)
- EF = Exposure frequency (days/year)
- ED = Exposure duration (years)
- AT = Averaging time (years)

For non-carcinogenic health risks/sub-chronic exposure, or when evaluating acute exposures or short-term events where the exposure duration aligns with the exposure averaging time, AT equals ED; therefore, AT and ED can be left out of Equation 2. In contrast, for carcinogenic risk/chronic exposure, the AT is equal to the estimated life expectancy.

The Rfc(s) represent the PM_2.5_ concentration values unlikely to cause adverse human health risks over a specified period. The Rfc(s) were derived from toxicological studies and are expressed in the same units as C_air_ (i.e., μg/m^3^). According to USEPA (62), if the HQ is less or equal to one (HQ ≤ 1), the concentration of PM_2.5_ (C_air_) is equal to or less than the Rfc. If the HQ is greater than one (HQ > 1), there is an increased likelihood of developing non-carcinogenic adverse health outcomes.

The CR was calculated using Equation 3 for each population sub-group to assess the carcinogenic health risks associated with exposure to PM_2.5_.

(3) $$\mathrm{CR}=\mathrm{IUR}\times C_{\mathrm{air}-\mathrm{adj}}$$

Where:

CR is the cancer risk, which represents the estimated probability of developing cancer over a lifetime due to exposure to PM_2.5_ through inhalation.

IUR is the inhalation unit risk, representing the increased risk of cancer per unit exposure to PM_2.5_ through inhalation. The IUR for PM_2.5_ is 0.008 μg/m^3^ (63).

A value greater than 1 × 10^−04^ (1 in 10,000) indicates a significant CR and may indicate a need for regulatory action or intervention to reduce exposure, as it exceeds the accepted risk threshold. On the other hand, a value less than 1 × 10^−06^ (1 in 1,000,000) indicates a negligible or “acceptable” risk. It can be ignored as it does not require regulatory action or interventions to reduce exposure (64).

## 3 Ethical considerations

The study was approved by the University of the Witwatersrand Human Research Ethics Committee [HREC] (No: HRECNMW21/05/09).

## 4 Results

### 4.1 Probabilistic human health risk assessment

#### 4.1.1 Adjusted PM_2.5_ concentrations for non-carcinogenic and carcinogenic health risks

The adjusted PM_2.5_ concentrations for non and carcinogenic health risks of the different population sub-groups in eMbalenhle are presented in Table 3. The 50th, 75th, and 95th percentile for adjusted PM_2.5_ concentrations for non-cancer risks was slightly higher in male and female adults compared to other subgroups due to the higher estimated ET. The 50^th^ percentile (median) adjusted PM_2.5_ concentrations for CR ranged from 0.4 μg/m^3^ (male and female toddlers and children) and 10.9 μg/m^3^ (male elders) due to the different estimates of the AT.

**Table 3:** The adjusted PM_2.5_ concentrations (μg/m^3^) for non and carcinogenic health risks.

Sub-groups	50th percentile		75th percentile		95th percentile	
	*NCR	**CR	*NCR	**CR	*NCR	**CR
Male toddlers	14.4	0.4	15.6	0.5	17.4	0.6
Female toddlers	14.5	0.4	15.6	0.4	17.4	0.5
Male children	14.0	2.2	15.3	2.6	17.2	3.2
Female children	14.1	1.9	15.3	2.2	17.2	2.7
Male adults	14.9	10.5	16.0	13.1	17.8	16.4
Female adults	14.9	9.6	16.0	12.0	17.7	15.1
Male elders	14.9	17.0	16.0	18.7	17.7	21.6
Female elders	14.9	15.7	16.0	17.3	17.7	20.0

*NCR: Non-Carcinogenic Health Risks.

**CR: Carcinogenic Health Risks.

Non-carcinogenic health risks based on PM_2.5_ annual reference standards are presented in Table 4. Based on the annual PM_2.5_ SA NAAQS, all population sub-groups in eMbalenhle showed 95th percentile HQ values below 1. Using the USEPA NAAQS, all population subgroups showed HQ values greater than 1, using the 50th, 75th and 95th percentiles of C_air-adj_. Throughout all the reference standards (WHO Guidelines, SA and US EPA NAAQS), the 50th, 75th and 95th HQ percentiles indicated varying health risks of exposure to PM_2.5_ eMbalenhle. The WHO Guidelines showed the highest potential to curb non-carcinogenic risks, followed by USEPA and the SA NAAQS. When estimating the hazard quotient (HQ) for the 95th percentile of the SA NAAQS, the risk of developing non-carcinogenic health effects was four times lower than WHO’s HQ. This difference indicates that using NAAQS to evaluate health risks could underestimate the risk for individuals exposed to PM_2.5_ in eMbalenhle.

**Table 4:** Non-carcinogenic risk assessment of PM_2.5_ among population sub-groups in eMbalenhle.

Population	Standard/guideline	Percentile	HQ
*All Population Sub-groups (Male and Female Toddlers, Children, Adults, and Elders)	SA NAAQS	50th	0.7
		75th	0.8
		95th	0.9
	USEPA	50th	1.7
		75th	1.8
		95th	2.0
	WHO	50th	3.0
		75th	3.2
		95th	3.5

*The computed HQ values for each subpopulation group are the same based on the NCR values presented in Table 3.

#### 4.1.2 Carcinogenic risk assessment of exposure to PM_2.5_ in eMbalenhle

**Figure 1:**
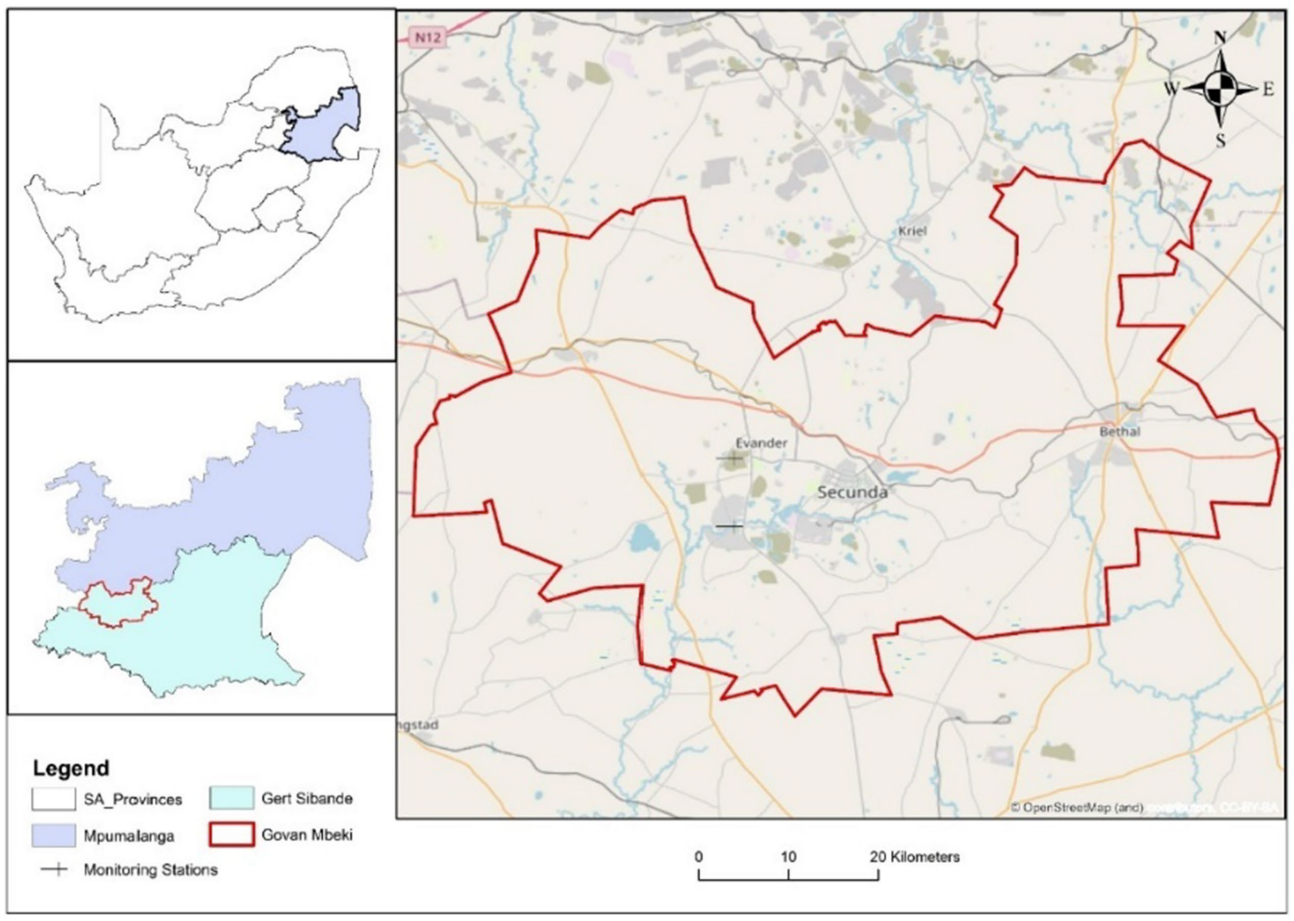
Maps showing the study area within the Country, Province, and District.

The results in Table 5 and Figure 1 report the CR for male and female age groups in eMbalenhle based on exposure to adjusted PM_2.5_ related to their characteristics. For both male and female elders, the CR was approximately 1 in 10, meaning that about 100,000 out of 1,000,000 exposed elders were at an increased risk of developing cancer over their lifetime due to PM_2.5_ exposure at the reported levels. Even for toddlers born and raised in eMbalenhle, the probability of developing cancer during their lifetime exceeds the critical 1 × 10^−04^ level.

**Table 5:** Carcinogenic human health risks of exposure to PM_2.5_.

Population sub-groups	50th-percentile	75th-percentile	95th-percentile
Male toddlers	3 × 10^−3^	4 × 10^−3^	4 × 10^−3^
Female toddlers	3 × 10^−3^	3 × 10^−3^	4 × 10^−3^
Male children	2 × 10^−2^	2 × 10^−2^	3 × 10^−2^
Female children	2 × 10^−2^	2 × 10^−2^	2 × 10^−2^
Male adults	8 × 10^−2^	10 × 10^−2^	1 × 10^−1^
Female adults	7 × 10^−2^	10 × 10^−2^	1 × 10^−1^
Male elders	1 × 10^−1^	2 × 10^−1^	2 × 10^−1^
Female elders	1 × 10^−1^	1 × 10^−1^	2 × 10^−1^

#### 4.1.3 Sensitivity analysis of model inputs based on non-cancer and cancer risks

**Figure 2:**
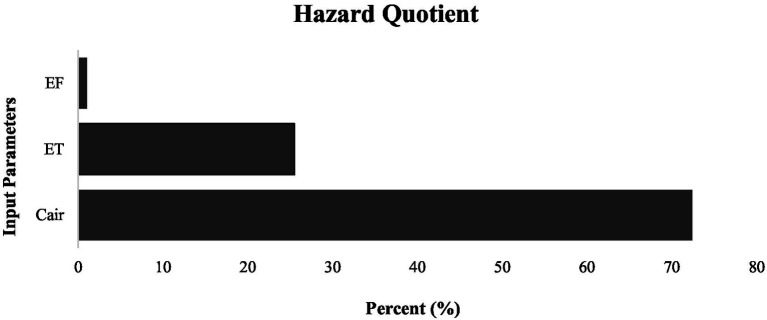
Sensitivity analysis of the parameters based on non-cancer risks.

**Figure 3:**
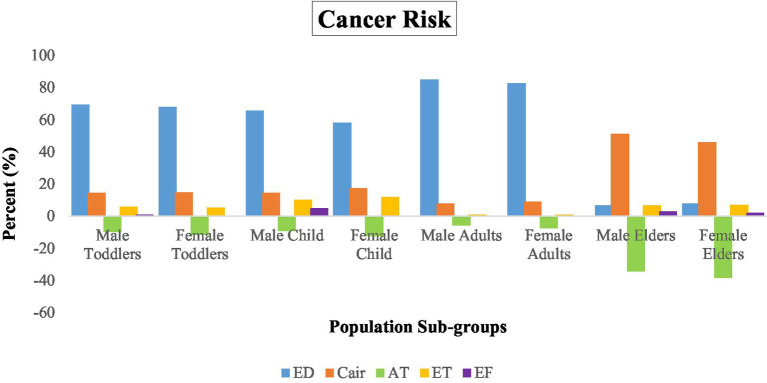
Sensitivity analysis of the input parameters based on cancer risks.

The contribution of input parameters to the variance in non-cancer risks is shown in Figures 2, 3. The variation of C_air_ showed the highest contribution to the variance in the non-cancer risk (72.4%, range of 57–87%), whereas on average, ET and EF contributed 25.5% (range of 11–41%) and 4% (range of 1–7%), respectively to the variance in non-cancer risks.

Figure 3 shows that the parameter sensitivity for the variance in CR for the older adult subgroups deviates from the other age groups. The highest contributions to the CR variance for the older adult were the C_air_ and the AT, whereas the ED showed the highest sensitivity for the other subgroups.

## 5 Discussion

This study reported an annual GM PM_2.5_ concentration of 17 μg/m^3^. Various PM_2.5_ sources, including traffic emissions, industrial activities, domestic cooking, space heating, waste-burning and dust from gold mine TSFs, could have contributed to the increased levels of PM_2.5_ in eMbalenhle. This annual concentration level was below the South African NAAQS of 20 μg/m^3^. However, it exceeded the WHO air quality guidelines and the USEPA NAAQS of 5 μg/m^3^ and 9 μg/m^3^, respectively. This indicated that although the local air quality standards are met, the exposed population may still experience significant health risks when the reported PM_2.5_ concentrations are compared with international reference standards. The exceedances of international standards from February 2022 to February 2023 indicated a potential public health concern, especially for vulnerable population sub-groups living close to sources of PM_2.5_ and gold mine TSFs. Although annual PM_2.5_ emissions reported in this study are slightly below the SA NAAQS, Millar et al. (65) that, for the past 10 years (2009–2019), the annual PM_2.5_ concentrations in eMbalenhle exceeded the SA NAAQS each year. The study used high-resolution data from the South African Weather Service (SAWS) air quality monitoring station.

Different countries have varying ambient PM_2.5_ NAAQSs. This is due to environmental, health, social, political and economic factors (66) affecting these countries. The WHO (51) provides guidelines for air quality, but countries adopt these recommendations differently based on their national circumstances. In most cases, developing countries may prioritize economic growth and industrialization over strict air quality standards to avoid limiting industrial expansion (67). In contrast, developed countries may impose more stringent regulations to protect public health and the environment. Moreover, countries with advanced air quality management and monitoring technologies may set and enforce stricter NAAQS (68). Countries with less technological capacity may set more lenient standards due to challenges in air quality monitoring (69).

For the probalistic HHRA, the PM_2.5_ concentrations were adjusted to reflect an accurate estimation of an equivalent full-day exposure over 1 year in the eMbalenhle population. The results showed that the median (50th percentile) for non-carcinogenic risk was low (below 1) across all population subgroups based on SA NAAQS. However, compared to the more restrictive USEPA and WHO air quality guidelines, even the median HQ values demonstrated a substantially increased risk with HQ values of 1.5 and 2.8, respectively. Amnuaylojaroen and Parasin (70) computed higher HQ values than this study. This is partly because they used RfC of 35 μg/m^3^ to calculate the HQ and worst-case assumptions regarding the risk parameters. The mean HQs were 2.93, 2.59, 2.28, 1.88, and 1.26 for newborns, toddlers, young children, school-age children, and adolescents, respectively.

Although the WHO air quality guidelines are widely adopted and used in many countries, they are not absolute and can be overly simplistic. First, the guidelines fail to account for cumulative risks and variability in individual susceptibility. Second, the guidelines fail to account for risk tolerance, which can vary between populations and regulatory bodies. Given this, there is a greater need to develop more robust and advanced approaches that factor in these issues to reflect real-world exposures accurately and avoid underestimating or overestimating risk.

For the cancer risk (CR), results showed values greater than 1.0 × 10^−4^ (1 in 10,000) for all population subgroups, which is generally considered significant, calling for stricter regulatory action and interventions to prevent further exposure. For example, the 50th, 75th and 95th percentile values indicated significant CRs (all greater than 1.0 × 10^−4^) across all population sub-groups. The CR became more significant in male and female elders. This result is similar to Wu et al. (71), who found the CR more significant in male elders. In contrast, Stapelfeld et al. (72) found that females were more at risk of developing cancer than males. In our study, the slightly higher CR for men was due to the lower life expectancy than women, but this difference is not statistically significant. Furthermore, males have different hormonal profiles compared to females (73) and as they age, the risk of developing cancer increases (74).

The female children showed the least chance of developing CR because of PM_2.5_ exposure compared to the other groups. Our estimations were determined by the ED/AT ratio, which was the lowest for toddlers and the highest for elders. Since toddlers and children are young, their risk for developing cancer is reduced because they have less accumulated genetic mutations (75). Contrary to this, Siegel et al. (76) observed that pediatric cancers were more pronounced in male children than in females.

The sensitivity analysis indicated that C_air_ and ET had the most significant impact on non-cancer risks. Since C_air_ is measured, the estimates for ET are key for estimating the extent of non-cancer risk. These findings call for regulatory action and policies to reduce exposures, especially to vulnerable groups. Strategies could include promulgating stringent emission standards for petrochemical industries, power plants, coal mines and gold mines. Inside the community, there should be an advocation for the transition to cleaner fuels, energy-efficient cookstoves and renewable energy sources to mitigate combustion-related PM_2.5_ emissions (77). Other strategies could include the spatial resolution of air quality monitoring in the area by adopting low-cost sensors (78), issuing early warnings for high pollution days (79), and raising public awareness to empower communities to take protective measures. On the other hand, the ED was the most sensitive parameter for the CR due to the assumed uniform distribution in the adult subgroup. For the older adult, where the ED/AT ratio is close to one, the variation in C_air_ becomes more significant for the estimates of the CR. In a similar study, Amoatey et al. (80) observed that long durations of exposure to ambient PM_2.5_ increased adverse health outcomes amongst the Roman population.

Applying the probabilistic HHRA model may be more complex than the deterministic approach (81). The determinist HHRA approach uses fixed point estimates for input variables (ED, ET, EF, AT) (82). Often, the maximum values are used, resulting in worst-case assessments. In contrast, the probabilistic HHRA uses probability distributions for input variables to account for variability and uncertainty (83). Amnuaylojaroen and Parasin (70) applied a deterministic risk assessment model to assess the health impacts of exposure to PM_2.5_ in different age groups of children in Northern Thailand. Compared to the probabilistic HHRA model that was applied in this study, their model considered the body weight and inhalation rate as variables, as also in Morakinyo et al. (84). The study also postulated that PM_2.5_ exposure might affect children differently depending on gender, with males at a higher risk than females in adolescence (70).

## 6 Conclusion

The findings of the study demonstrated that the SA NAAQS may not adequately protect public health as it may underestimate the risks associated with PM_2.5_. To protect public health, there is a need for the government to adopt more stringent standards and align them closely with international standards, including the WHO guidelines.

Using a probabilistic human health risk assessment (HHRA) approach, the study revealed that non-carcinogenic risks were low when benchmarked against the SA NAAQS, but substantially elevated when compared with international guidelines. Vulnerable groups, particularly newborns and toddlers, exhibited higher hazard quotients (HQs), underscoring their increased susceptibility. For cancer risks (CR), results exceeded the generally acceptable threshold of 1 in 10,000 across all population groups, particularly affecting the older adult.

PM_2.5_ concentrations were the most significant factor in increased non-cancer health problems in all sub-population groups. This emphasizes the need for control measures to reduce PM_2.5_ concentrations, including the maintenance of TSFs and increasing monitoring in hot spot areas. The ED was the most critical factor for the cancer risks, followed by the air concentration for all population sub-groups except for the older adult. Strategies to reduce the ED and EF, such as limiting outdoor activities during high pollution events could further minimize health risks associated with exposure to PM_2.5_. The sensitivity analysis indicated that ambient PM_2.5_ concentration (C_air_) and exposure time (ET) had the most influence on non-cancer risk estimates, while exposure duration (ED) was key for cancer risks. These observations indicate the urgent need for targeted interventions to minimize exposure, especially among vulnerable populations. Policy actions could include tighter emissions controls, community-level interventions to promote dust control alternatives, TSF management strategies, and expanded air quality monitoring networks using low-cost sensors.

Considering the above, this study calls for an urgent review of the current South African NAAQS. However, policymakers should strike a good and functional balance between economic imperatives and public health outcomes. Even though the WHO provides global benchmarks, countries can adopt these to meet their socio-economic context, technological feasibility and regulatory frameworks.

## 7 Study limitations

The LCM measurement results over the year represent the temporal variations; however, spatial variations were not covered, limiting the representativeness of the PM_2.5_ concentration data for the general population of eMbalenhle. In addition to this measured parameter (C_air_), the HQ and CR risk equations, distribution and values for all other parameters, except life expectancy, were only estimates and not based on actual data or information from the community. In-depth population studies are needed to collect more relevant information, especially for the highest sensitivity risk parameters. Therefore, this study’s HQ and CR probability distributions can only be considered to flag potential health risks for the eMbalenhle residents. Lastly, this study made assumptions for the ED and EF for all population subgroups. This can limit the reliability and generalizability of the findings, as these parameters may not accurately reflect the diverse exposure patterns across the population subgroups. Uniform distribution patterns and assumptions of uniform exposure may overlook important activity data, including age-specific behaviours and varying susceptibility. This could lead to potential underestimation or overestimation of risks.

## Figures and Tables

**Figure A1:**
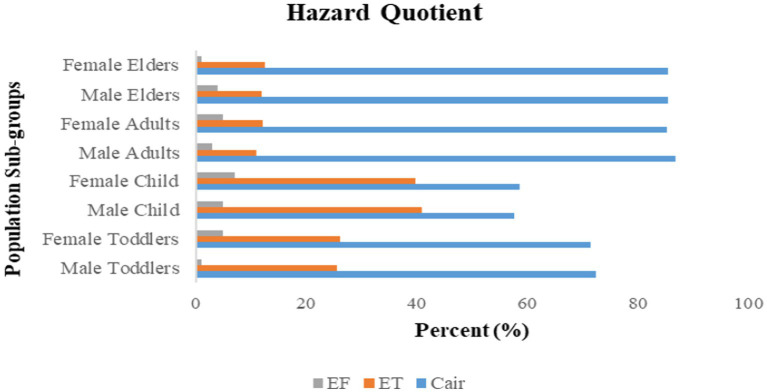
Sensitivity analysis of the parameters based on non-cancer risks in eMbalenhle.

## Data Availability

The raw data supporting the conclusions of this article will be made available by the authors without undue reservation.
